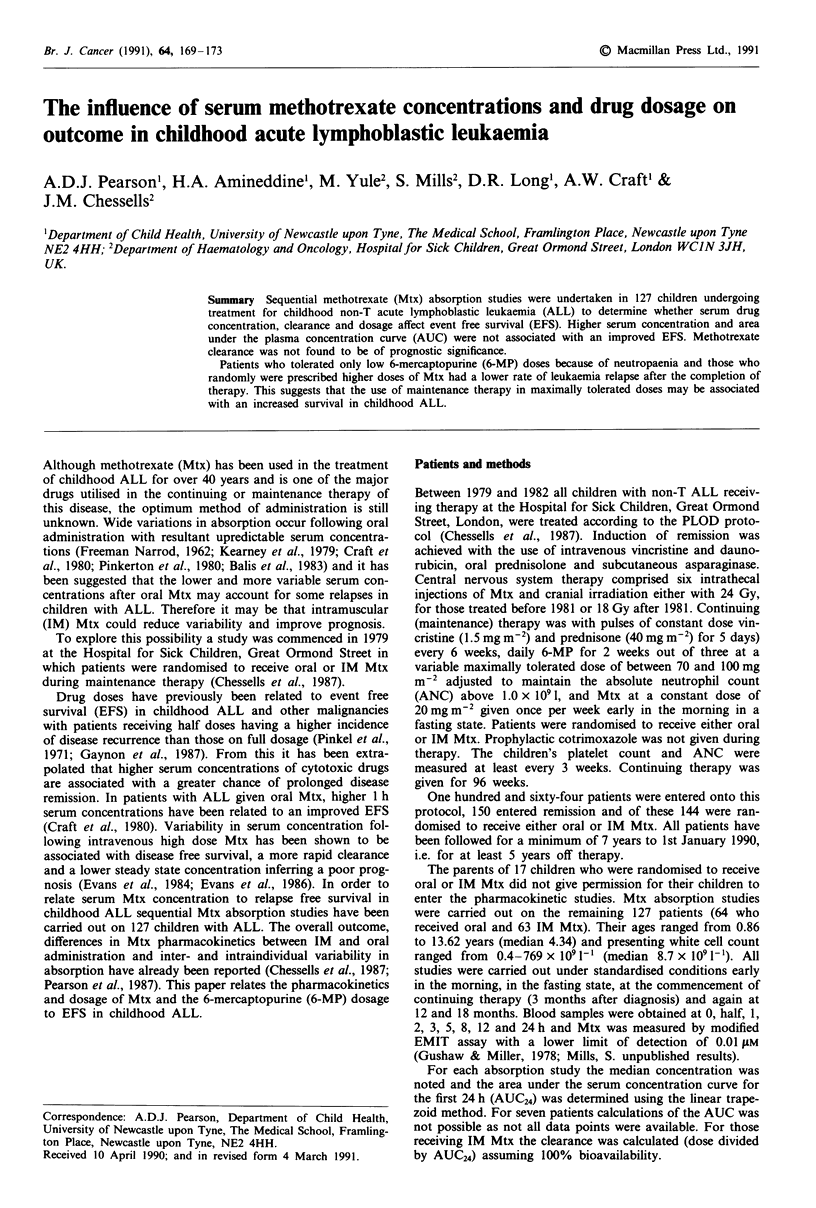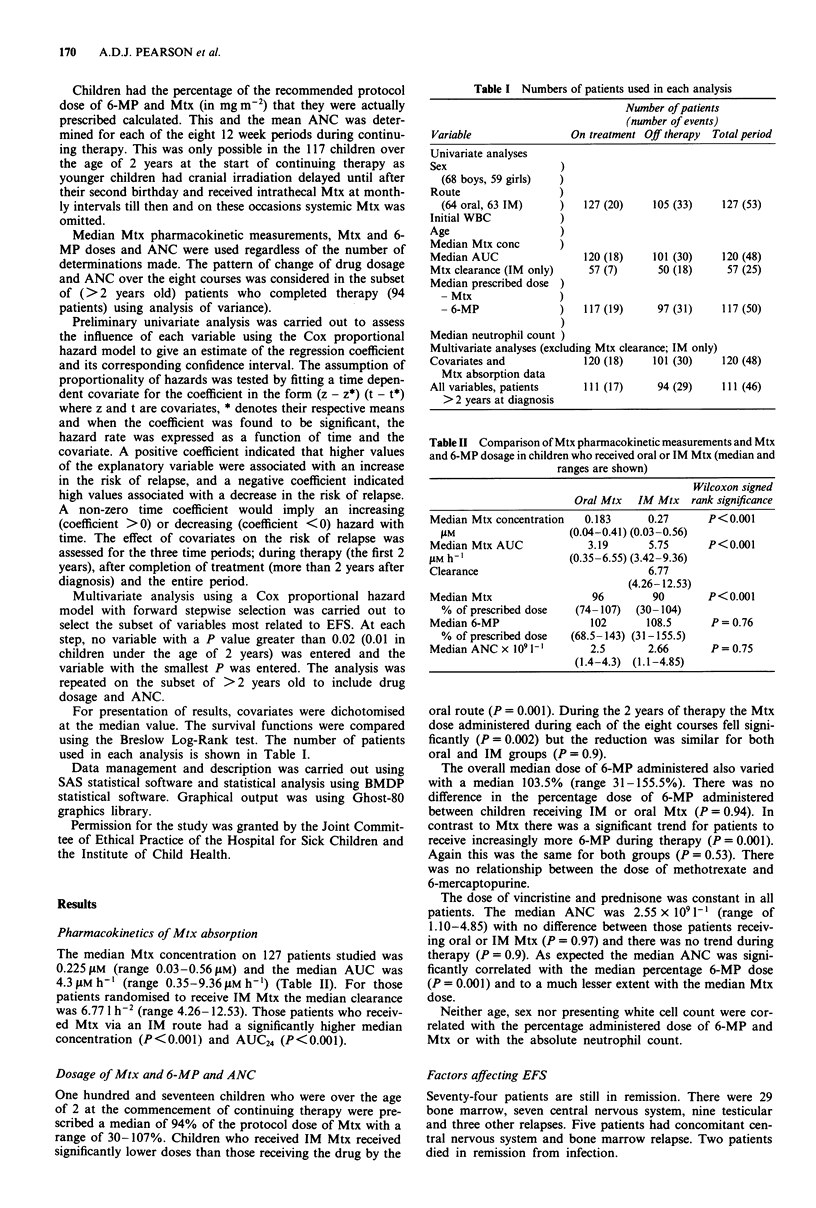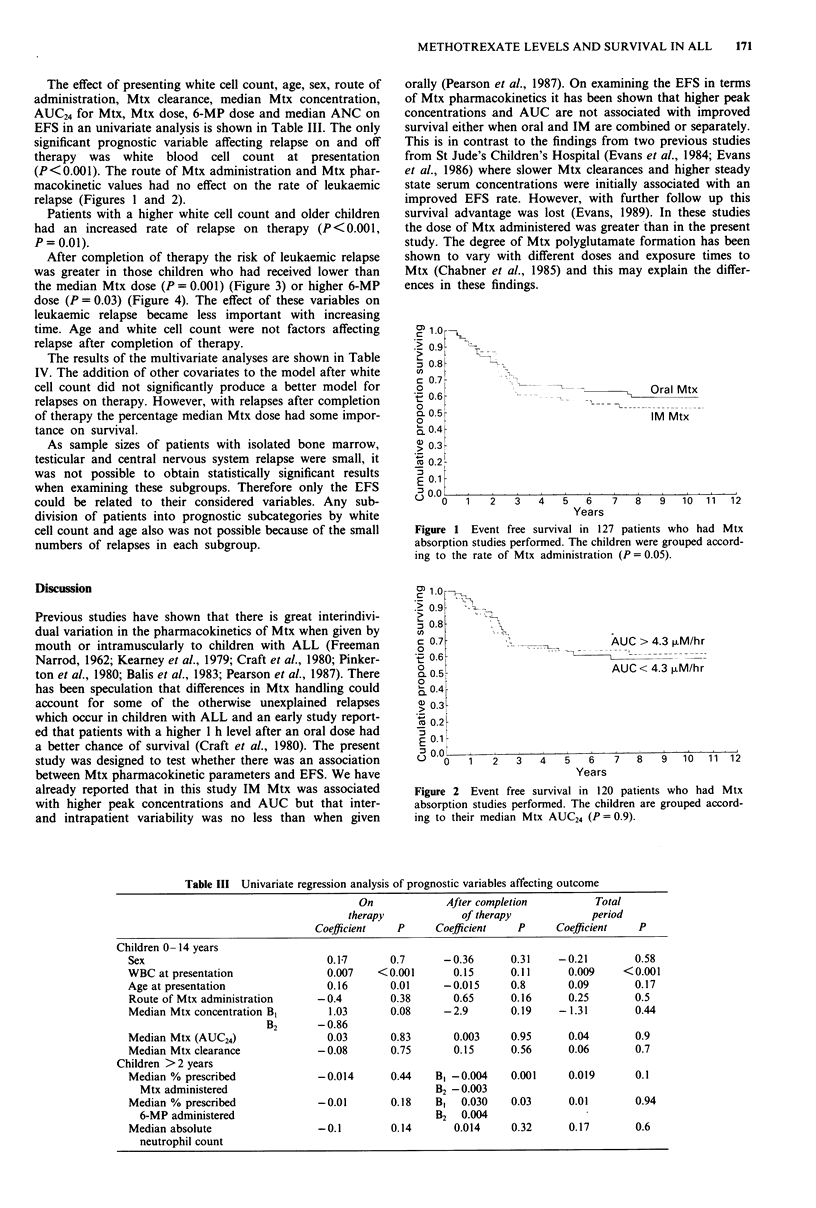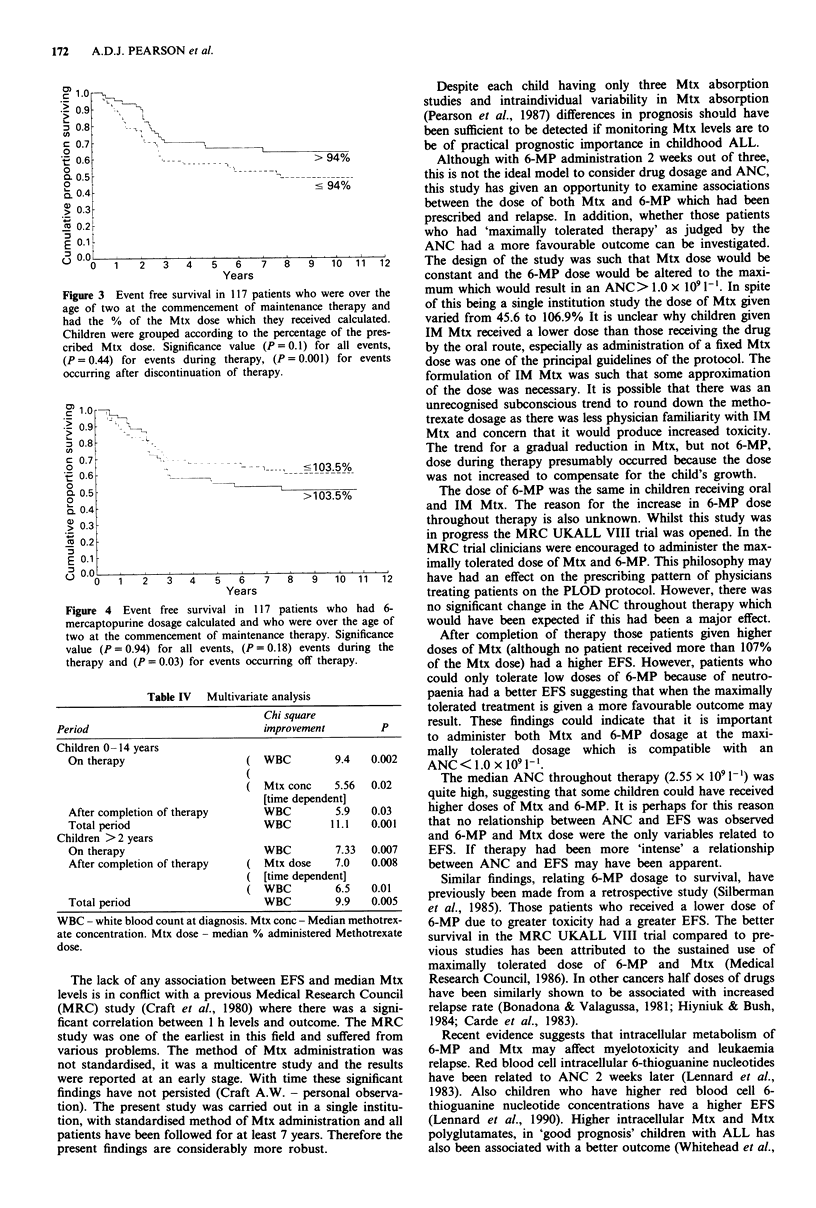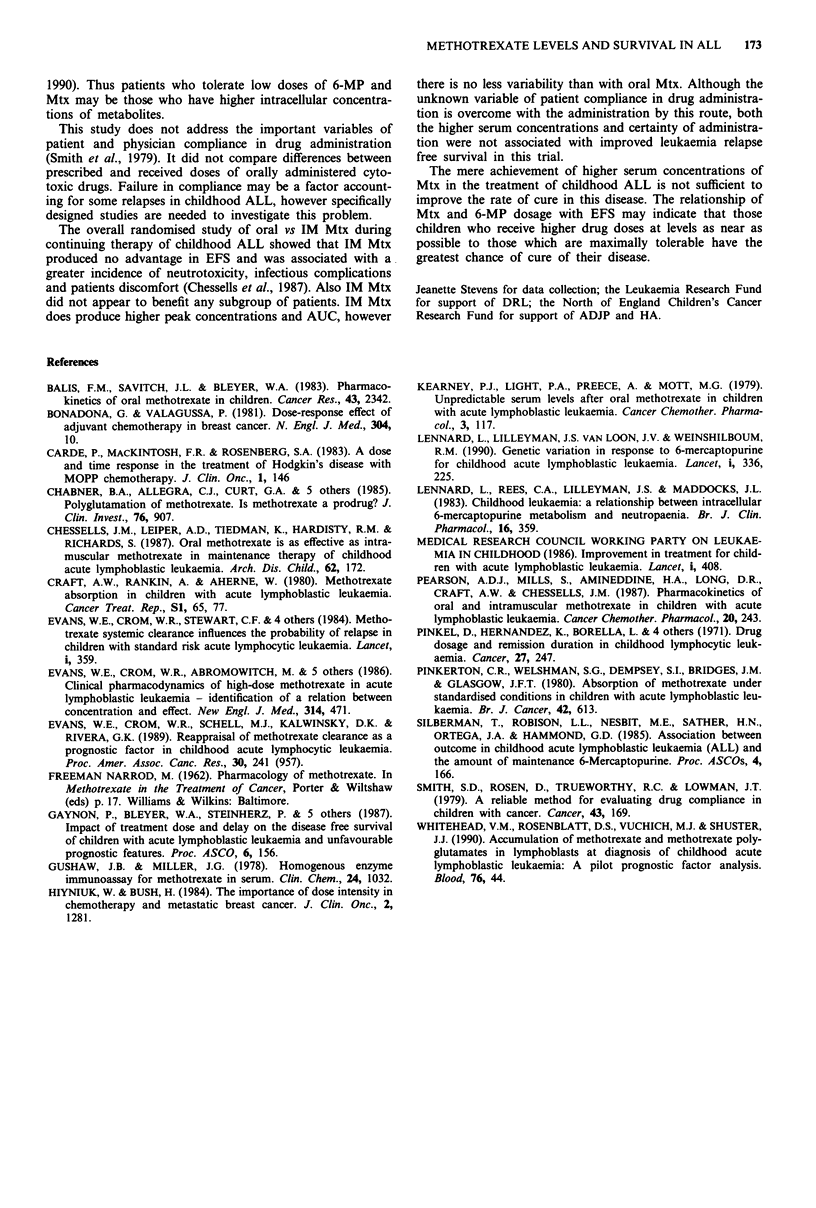# The influence of serum methotrexate concentrations and drug dosage on outcome in childhood acute lymphoblastic leukaemia.

**DOI:** 10.1038/bjc.1991.263

**Published:** 1991-07

**Authors:** A. D. Pearson, H. A. Amineddine, M. Yule, S. Mills, D. R. Long, A. W. Craft, J. M. Chessells

**Affiliations:** Department of Child Health, University of Newcastle upon Tyne, Medical School, UK.

## Abstract

Sequential methotrexate (Mtx) absorption studies were undertaken in 127 children undergoing treatment for childhood non-T acute lymphoblastic leukaemia (ALL) to determine whether serum drug concentration, clearance and dosage affect event free survival (EFS). Higher serum concentration and area under the plasma concentration curve (AUC) were not associated with an improved EFS. Methotrexate clearance was not found to be of prognostic significance. Patients who tolerated only low 6-mercaptopurine (6-MP) doses because of neutropaenia and those who randomly were prescribed higher doses of Mtx had a lower rate of leukaemia relapse after the completion of therapy. This suggests that the use of maintenance therapy in maximally tolerated doses may be associated with an increased survival in childhood ALL.


*Br. J. Cancer* (1991), **64**, 169–173 © Macmillan Press Ltd., 1991

### The influence of serum methotrexate concentrations and drug dosage on outcome in childhood acute lymphoblastic leukaemia

A.D.J. Pearson[1], H.A. Amineddine[1], M. Yule[2], S. Mills[2], D.R. Long[1], A.W. Craft[1] & J.M. Chessells[2]

[1]*Department of Child Health, University of Newcastle upon Tyne, The Medical School, Framlington Place, Newcastle upon Tyne NE2 4HH;* [2]*Department of Haematology and Oncology, Hospital for Sick Children, Great Ormond Street, London WC1N 3JH, UK.*

**Summary** Sequential methotrexate (Mtx) absorption studies were undertaken in 127 children undergoing treatment for childhood non-T acute lymphoblastic leukaemia (ALL) to determine whether serum drug concentration, clearance and dosage affect event free survival (EFS). Higher serum concentration and area under the plasma concentration curve (AUC) were not associated with an improved EFS. Methotrexate clearance was not found to be of prognostic significance.

Patients who tolerated only low 6-mercaptopurine (6-MP) doses because of neutropaenia and those who randomly were prescribed higher doses of Mtx had a lower rate of leukaemia relapse after the completion of therapy. This suggests that the use of maintenance therapy in maximally tolerated doses may be associated with an increased survival in childhood ALL.

Although methotrexate (Mtx) has been used in the treatment of childhood ALL for over 40 years and is one of the major drugs utilised in the continuing or maintenance therapy of this disease, the optimum method of administration is still unknown. Wide variations in absorption occur following oral administration with resultant upredictable serum concentrations (Freeman Narrod, 1962; Kearney *et al.*, 1979; Craft *et al.*, 1980; Pinkerton *et al.*, 1980; Balis *et al.*, 1983) and it has been suggested that the lower and more variable serum concentrations after oral Mtx may account for some relapses in children with ALL. Therefore it may be that intramuscular (IM) Mtx could reduce variability and improve prognosis.

To explore this possibility a study was commenced in 1979 at the Hospital for Sick Children, Great Ormond Street in which patients were randomised to receive oral or IM Mtx during maintenance therapy (Chessells *et al.*, 1987).

Drug doses have previously been related to event free survival (EFS) in childhood ALL and other malignancies with patients receiving half doses having a higher incidence of disease recurrence than those on full dosage (Pinkel *et al.*, 1971; Gaynon *et al.*, 1987). From this it has been extrapolated that higher serum concentrations of cytotoxic drugs are associated with a greater chance of prolonged disease remission. In patients with ALL given oral Mtx, higher 1 h serum concentrations have been related to an improved EFS (Craft *et al.*, 1980). Variability in serum concentration following intravenous high dose Mtx has been shown to be associated with disease free survival, a more rapid clearance and a lower steady state concentration inferring a poor prognosis (Evans *et al.*, 1984; Evans *et al.*, 1986). In order to relate serum Mtx concentration to relapse free survival in childhood ALL sequential Mtx absorption studies have been carried out on 127 children with ALL. The overall outcome, differences in Mtx pharmacokinetics between IM and oral administration and inter- and intraindividual variability in absorption have already been reported (Chessells *et al.*, 1987; Pearson *et al.*, 1987). This paper relates the pharmacokinetics and dosage of Mtx and the 6-mercaptopurine (6-MP) dosage to EFS in childhood ALL.

Correspondence: A.D.J. Pearson, Department of Child Health, University of Newcastle upon Tyne, The Medical School, Framlington Place, Newcastle upon Tyne, NE2 4HH.
Received 10 April 1990; and in revised form 4 March 1991.

### Patients and methods

Between 1979 and 1982 all children with non-T ALL receiving therapy at the Hospital for Sick Children, Great Ormond Street, London, were treated according to the PLOD protocol (Chessells *et al.*, 1987). Induction of remission was achieved with the use of intravenous vincristine and daunorubicin, oral prednisolone and subcutaneous asparaginase. Central nervous system therapy comprised six intrathecal injections of Mtx and cranial irradiation either with 24 Gy, for those treated before 1981 or 18 Gy after 1981. Continuing (maintenance) therapy was with pulses of constant dose vincristine ($1.5\,mg\,m^{-2}$) and prednisone ($40\,mg\,m^{-2}$) for 5 days) every 6 weeks, daily 6-MP for 2 weeks out of three at a variable maximally tolerated dose of between 70 and 100 mg $m^{-2}$ adjusted to maintain the absolute neutrophil count (ANC) above $1.0 \times 10^9\,l$, and Mtx at a constant dose of $20\,mg\,m^{-2}$ given once per week early in the morning in a fasting state. Patients were randomised to receive either oral or IM Mtx. Prophylactic cotrimoxazole was not given during therapy. The children's platelet count and ANC were measured at least every 3 weeks. Continuing therapy was given for 96 weeks.

One hundred and sixty-four patients were entered onto this protocol, 150 entered remission and of these 144 were randomised to receive either oral or IM Mtx. All patients have been followed for a minimum of 7 years to 1st January 1990, i.e. for at least 5 years off therapy.

The parents of 17 children who were randomised to receive oral or IM Mtx did not give permission for their children to enter the pharmacokinetic studies. Mtx absorption studies were carried out on the remaining 127 patients (64 who received oral and 63 IM Mtx). Their ages ranged from 0.86 to 13.62 years (median 4.34) and presenting white cell count ranged from $0.4–769 \times 10^9\,l^{-1}$ (median $8.7 \times 10^9\,l^{-1}$). All studies were carried out under standardised conditions early in the morning, in the fasting state, at the commencement of continuing therapy (3 months after diagnosis) and again at 12 and 18 months. Blood samples were obtained at 0, half, 1, 2, 3, 5, 8, 12 and 24 h and Mtx was measured by modified EMIT assay with a lower limit of detection of 0.01 $\mu$M (Gushaw & Miller, 1978; Mills, S. unpublished results).

For each absorption study the median concentration was noted and the area under the serum concentration curve for the first 24 h ($AUC_{24}$) was determined using the linear trapezoid method. For seven patients calculations of the AUC was not possible as not all data points were available. For those receiving IM Mtx the clearance was calculated (dose divided by $AUC_{24}$) assuming 100% bioavailability.

Children had the percentage of the recommended protocol dose of 6-MP and Mtx (in mg $m^{-2}$) that they were actually prescribed calculated. This and the mean ANC was determined for each of the eight 12 week periods during continuing therapy. This was only possible in the 117 children over the age of 2 years at the start of continuing therapy as younger children had cranial irradiation delayed until after their second birthday and received intrathecal Mtx at monthly intervals till then and on these occasions systemic Mtx was omitted.

Median Mtx pharmacokinetic measurements, Mtx and 6-MP doses and ANC were used regardless of the number of determinations made. The pattern of change of drug dosage and ANC over the eight courses was considered in the subset of (>2 years old) patients who completed therapy (94 patients) using analysis of variance).

Preliminary univariate analysis was carried out to assess the influence of each variable using the Cox proportional hazard model to give an estimate of the regression coefficient and its corresponding confidence interval. The assumption of proportionality of hazards was tested by fitting a time dependent covariate for the coefficient in the form $(z - z^*)(t - t^*)$ where z and t are covariates, * denotes their respective means and when the coefficient was found to be significant, the hazard rate was expressed as a function of time and the covariate. A positive coefficient indicated that higher values of the explanatory variable were associated with an increase in the risk of relapse, and a negative coefficient indicated high values associated with a decrease in the risk of relapse. A non-zero time coefficient would imply an increasing (coefficient >0) or decreasing (coefficient <0) hazard with time. The effect of covariates on the risk of relapse was assessed for the three time periods; during therapy (the first 2 years), after completion of treatment (more than 2 years after diagnosis) and the entire period.

Multivariate analysis using a Cox proportional hazard model with forward stepwise selection was carried out to select the subset of variables most related to EFS. At each step, no variable with a *P* value greater than 0.02 (0.01 in children under the age of 2 years) was entered and the variable with the smallest *P* was entered. The analysis was repeated on the subset of >2 years old to include drug dosage and ANC.

For presentation of results, covariates were dichotomised at the median value. The survival functions were compared using the Breslow Log-Rank test. The number of patients used in each analysis is shown in Table I.

Data management and description was carried out using SAS statistical software and statistical analysis using BMDP statistical software. Graphical output was using Ghost-80 graphics library.

Permission for the study was granted by the Joint Committee of Ethical Practice of the Hospital for Sick Children and the Institute of Child Health.

**Table I** Numbers of patients used in each analysis

| *Variable* | *On treatment* | *Off therapy* | *Total period* |
|---|---|---|---|
| | *Number of patients (number of events)* | | |
| Univariate analyses | | | |
| Sex ) | | | |
| (68 boys, 59 girls) ) | | | |
| Route ) | | | |
| (64 oral, 63 IM) ) | 127 (20) | 105 (33) | 127 (53) |
| Initial WBC ) | | | |
| Age ) | | | |
| Median Mtx conc ) | | | |
| Median AUC | 120 (18) | 101 (30) | 120 (48) |
| Mtx clearance (IM only) | 57 (7) | 50 (18) | 57 (25) |
| Median prescribed dose ) | | | |
| – Mtx ) | | | |
| – 6-MP ) | 117 (19) | 97 (31) | 117 (50) |
| ) | | | |
| Median neutrophil count ) | | | |
| Multivariate analyses (excluding Mtx clearance; IM only) | | | |
| Covariates and Mtx absorption data | 120 (18) | 101 (30) | 120 (48) |
| All variables, patients >2 years at diagnosis | 111 (17) | 94 (29) | 111 (46) |

**Table II** Comparison of Mtx pharmacokinetic measurements and Mtx and 6-MP dosage in children who received oral or IM Mtx (median and ranges are shown)

| | *Oral Mtx* | *IM Mtx* | *Wilcoxon signed rank significance* |
|---|---|---|---|
| Median Mtx concentration μM | 0.183 (0.04–0.41) | 0.27 (0.03–0.56) | $P<0.001$ |
| Median Mtx AUC μM $h^{-1}$ | 3.19 (0.35–6.55) | 5.75 (3.42–9.36) | $P<0.001$ |
| Clearance | | 6.77 (4.26–12.53) | |
| Median Mtx % of prescribed dose | 96 (74–107) | 90 (30–104) | $P<0.001$ |
| Median 6-MP % of prescribed dose | 102 (68.5–143) | 108.5 (31–155.5) | $P=0.76$ |
| Median ANC $\times 10^9 l^{-1}$ | 2.5 (1.4–4.3) | 2.66 (1.1–4.85) | $P=0.75$ |

### Results

### *Pharmacokinetics of Mtx absorption*

The median Mtx concentration on 127 patients studied was 0.225 μM (range 0.03–0.56 μM) and the median AUC was 4.3 μM $h^{-1}$ (range 0.35–9.36 μM $h^{-1}$) (Table II). For those patients randomised to receive IM Mtx the median clearance was 6.77 l $h^{-2}$ (range 4.26–12.53). Those patients who received Mtx via an IM route had a significantly higher median concentration ($P<0.001$) and $AUC_{24}$ ($P<0.001$).

### *Dosage of Mtx and 6-MP and ANC*

One hundred and seventeen children who were over the age of 2 at the commencement of continuing therapy were prescribed a median of 94% of the protocol dose of Mtx with a range of 30–107%. Children who received IM Mtx received significantly lower doses than those receiving the drug by the oral route ($P=0.001$). During the 2 years of therapy the Mtx dose administered during each of the eight courses fell significantly ($P=0.002$) but the reduction was similar for both oral and IM groups ($P=0.9$).

The overall median dose of 6-MP administered also varied with a median 103.5% (range 31–155.5%). There was no difference in the percentage dose of 6-MP administered between children receiving IM or oral Mtx ($P=0.94$). In contrast to Mtx there was a significant trend for patients to receive increasingly more 6-MP during therapy ($P=0.001$). Again this was the same for both groups ($P=0.53$). There was no relationship between the dose of methotrexate and 6-mercaptopurine.

The dose of vincristine and prednisone was constant in all patients. The median ANC was $2.55 \times 10^9 l^{-1}$ (range of 1.10–4.85) with no difference between those patients receiving oral or IM Mtx ($P=0.97$) and there was no trend during therapy ($P=0.9$). As expected the median ANC was significantly correlated with the median percentage 6-MP dose ($P=0.001$) and to a much lesser extent with the median Mtx dose.

Neither age, sex nor presenting white cell count were correlated with the percentage administered dose of 6-MP and Mtx or with the absolute neutrophil count.

### *Factors affecting EFS*

Seventy-four patients are still in remission. There were 29 bone marrow, seven central nervous system, nine testicular and three other relapses. Five patients had concomitant central nervous system and bone marrow relapse. Two patients died in remission from infection.

The effect of presenting white cell count, age, sex, route of administration, Mtx clearance, median Mtx concentration, $AUC_{24}$ for Mtx, Mtx dose, 6-MP dose and median ANC on EFS in an univariate analysis is shown in Table III. The only significant prognostic variable affecting relapse on and off therapy was white blood cell count at presentation ($P < 0.001$). The route of Mtx administration and Mtx pharmacokinetic values had no effect on the rate of leukaemic relapse (Figures 1 and 2).

Patients with a higher white cell count and older children had an increased rate of relapse on therapy ($P < 0.001$, $P = 0.01$).

After completion of therapy the risk of leukaemic relapse was greater in those children who had received lower than the median Mtx dose ($P = 0.001$) (Figure 3) or higher 6-MP dose ($P = 0.03$) (Figure 4). The effect of these variables on leukaemic relapse became less important with increasing time. Age and white cell count were not factors affecting relapse after completion of therapy.

The results of the multivariate analyses are shown in Table IV. The addition of other covariates to the model after white cell count did not significantly produce a better model for relapses on therapy. However, with relapses after completion of therapy the percentage median Mtx dose had some importance on survival.

As sample sizes of patients with isolated bone marrow, testicular and central nervous system relapse were small, it was not possible to obtain statistically significant results when examining these subgroups. Therefore only the EFS could be related to their considered variables. Any subdivision of patients into prognostic subcategories by white cell count and age also was not possible because of the small numbers of relapses in each subgroup.

### Discussion

Previous studies have shown that there is great interindividual variation in the pharmacokinetics of Mtx when given by mouth or intramuscularly to children with ALL (Freeman Narrod, 1962; Kearney *et al.*, 1979; Craft *et al.*, 1980; Pinkerton *et al.*, 1980; Balis *et al.*, 1983; Pearson *et al.*, 1987). There has been speculation that differences in Mtx handling could account for some of the otherwise unexplained relapses which occur in children with ALL and an early study reported that patients with a higher 1 h level after an oral dose had a better chance of survival (Craft *et al.*, 1980). The present study was designed to test whether there was an association between Mtx pharmacokinetic parameters and EFS. We have already reported that in this study IM Mtx was associated with higher peak concentrations and AUC but that inter- and intrapatient variability was no less than when given orally (Pearson *et al.*, 1987). On examining the EFS in terms of Mtx pharmacokinetics it has been shown that higher peak concentrations and AUC are not associated with improved survival either when oral and IM are combined or separately. This is in contrast to the findings from two previous studies from St Jude's Children's Hospital (Evans *et al.*, 1984; Evans *et al.*, 1986) where slower Mtx clearances and higher steady state serum concentrations were initially associated with an improved EFS rate. However, with further follow up this survival advantage was lost (Evans, 1989). In these studies the dose of Mtx administered was greater than in the present study. The degree of Mtx polyglutamate formation has been shown to vary with different doses and exposure times to Mtx (Chabner *et al.*, 1985) and this may explain the differences in these findings.

**Figure 1** Event free survival in 127 patients who had Mtx absorption studies performed. The children were grouped according to the rate of Mtx administration ($P = 0.05$).

**Figure 2** Event free survival in 120 patients who had Mtx absorption studies performed. The children are grouped according to their median Mtx $AUC_{24}$ ($P = 0.9$).

**Table III** Univariate regression analysis of prognostic variables affecting outcome

| | *On therapy* Coefficient | P | *After completion of therapy* Coefficient | P | *Total period* Coefficient | P |
|---|---|---|---|---|---|---|
| Children 0–14 years | | | | | | |
| Sex | 0.17 | 0.7 | −0.36 | 0.31 | −0.21 | 0.58 |
| WBC at presentation | 0.007 | <0.001 | 0.15 | 0.11 | 0.009 | <0.001 |
| Age at presentation | 0.16 | 0.01 | −0.015 | 0.8 | 0.09 | 0.17 |
| Route of Mtx administration | −0.4 | 0.38 | 0.65 | 0.16 | 0.25 | 0.5 |
| Median Mtx concentration $B_1$ | 1.03 | 0.08 | −2.9 | 0.19 | −1.31 | 0.44 |
| $B_2$ | −0.86 | | | | | |
| Median Mtx ($AUC_{24}$) | 0.03 | 0.83 | 0.003 | 0.95 | 0.04 | 0.9 |
| Median Mtx clearance | −0.08 | 0.75 | 0.15 | 0.56 | 0.06 | 0.7 |
| Children >2 years | | | | | | |
| Median % prescribed Mtx administered | −0.014 | 0.44 | $B_1$ −0.004<br>$B_2$ −0.003 | 0.001 | 0.019 | 0.1 |
| Median % prescribed 6-MP administered | −0.01 | 0.18 | $B_1$ 0.030<br>$B_2$ 0.004 | 0.03 | 0.01 | 0.94 |
| Median absolute neutrophil count | −0.1 | 0.14 | 0.014 | 0.32 | 0.17 | 0.6 |

**Figure 3** Event free survival in 117 patients who were over the age of two at the commencement of maintenance therapy and had the % of the Mtx dose which they received calculated. Children were grouped according to the percentage of the prescribed Mtx dose. Significance value ($P = 0.1$) for all events, ($P = 0.44$) for events during therapy, ($P = 0.001$) for events occurring after discontinuation of therapy.

**Figure 4** Event free survival in 117 patients who had 6-mercaptopurine dosage calculated and who were over the age of two at the commencement of maintenance therapy. Significance value ($P = 0.94$) for all events, ($P = 0.18$) events during the therapy and ($P = 0.03$) for events occurring off therapy.

**Table IV** Multivariate analysis

| *Period* | | *Chi square improvement* | *P* |
|---|---|---|---|
| Children 0–14 years | | | |
| On therapy | ( WBC | 9.4 | 0.002 |
| | ( | | |
| | ( Mtx conc [time dependent] | 5.56 | 0.02 |
| After completion of therapy | WBC | 5.9 | 0.03 |
| Total period | WBC | 11.1 | 0.001 |
| Children >2 years | | | |
| On therapy | WBC | 7.33 | 0.007 |
| After completion of therapy | ( Mtx dose | 7.0 | 0.008 |
| | ( [time dependent] | | |
| | ( WBC | 6.5 | 0.01 |
| Total period | WBC | 9.9 | 0.005 |

WBC – white blood count at diagnosis. Mtx conc – Median methotrexate concentration. Mtx dose – median % administered Methotrexate dose.

The lack of any association between EFS and median Mtx levels is in conflict with a previous Medical Research Council (MRC) study (Craft *et al.*, 1980) where there was a significant correlation between 1 h levels and outcome. The MRC study was one of the earliest in this field and suffered from various problems. The method of Mtx administration was not standardised, it was a multicentre study and the results were reported at an early stage. With time these significant findings have not persisted (Craft A.W. – personal observation). The present study was carried out in a single institution, with standardised method of Mtx administration and all patients have been followed for at least 7 years. Therefore the present findings are considerably more robust.

Despite each child having only three Mtx absorption studies and intraindividual variability in Mtx absorption (Pearson *et al.*, 1987) differences in prognosis should have been sufficient to be detected if monitoring Mtx levels are to be of practical prognostic importance in childhood ALL.

Although with 6-MP administration 2 weeks out of three, this is not the ideal model to consider drug dosage and ANC, this study has given an opportunity to examine associations between the dose of both Mtx and 6-MP which had been prescribed and relapse. In addition, whether those patients who had 'maximally tolerated therapy' as judged by the ANC had a more favourable outcome can be investigated. The design of the study was such that Mtx dose would be constant and the 6-MP dose would be altered to the maximum which would result in an ANC $> 1.0 \times 10^9 l^{-1}$. In spite of this being a single institution study the dose of Mtx given varied from 45.6 to 106.9% It is unclear why children given IM Mtx received a lower dose than those receiving the drug by the oral route, especially as administration of a fixed Mtx dose was one of the principal guidelines of the protocol. The formulation of IM Mtx was such that some approximation of the dose was necessary. It is possible that there was an unrecognised subconscious trend to round down the methotrexate dosage as there was less physician familiarity with IM Mtx and concern that it would produce increased toxicity. The trend for a gradual reduction in Mtx, but not 6-MP, dose during therapy presumably occurred because the dose was not increased to compensate for the child's growth.

The dose of 6-MP was the same in children receiving oral and IM Mtx. The reason for the increase in 6-MP dose throughout therapy is also unknown. Whilst this study was in progress the MRC UKALL VIII trial was opened. In the MRC trial clinicians were encouraged to administer the maximally tolerated dose of Mtx and 6-MP. This philosophy may have had an effect on the prescribing pattern of physicians treating patients on the PLOD protocol. However, there was no significant change in the ANC throughout therapy which would have been expected if this had been a major effect.

After completion of therapy those patients given higher doses of Mtx (although no patient received more than 107% of the Mtx dose) had a higher EFS. However, patients who could only tolerate low doses of 6-MP because of neutropaenia had a better EFS suggesting that when the maximally tolerated treatment is given a more favourable outcome may result. These findings could indicate that it is important to administer both Mtx and 6-MP dosage at the maximally tolerated dosage which is compatible with an ANC $< 1.0 \times 10^9 l^{-1}$.

The median ANC throughout therapy ($2.55 \times 10^9 l^{-1}$) was quite high, suggesting that some children could have received higher doses of Mtx and 6-MP. It is perhaps for this reason that no relationship between ANC and EFS was observed and 6-MP and Mtx dose were the only variables related to EFS. If therapy had been more 'intense' a relationship between ANC and EFS may have been apparent.

Similar findings, relating 6-MP dosage to survival, have previously been made from a retrospective study (Silberman *et al.*, 1985). Those patients who received a lower dose of 6-MP due to greater toxicity had a greater EFS. The better survival in the MRC UKALL VIII trial compared to previous studies has been attributed to the sustained use of maximally tolerated dose of 6-MP and Mtx (Medical Research Council, 1986). In other cancers half doses of drugs have been similarly shown to be associated with increased relapse rate (Bonadona & Valagussa, 1981; Hiyniuk & Bush, 1984; Carde *et al.*, 1983).

Recent evidence suggests that intracellular metabolism of 6-MP and Mtx may affect myelotoxicity and leukaemia relapse. Red blood cell intracellular 6-thioguanine nucleotides have been related to ANC 2 weeks later (Lennard *et al.*, 1983). Also children who have higher red blood cell 6-thioguanine nucleotide concentrations have a higher EFS (Lennard *et al.*, 1990). Higher intracellular Mtx and Mtx polyglutamates, in 'good prognosis' children with ALL has also been associated with a better outcome (Whitehead *et al.*,

1990). Thus patients who tolerate low doses of 6-MP and Mtx may be those who have higher intracellular concentrations of metabolites.

This study does not address the important variables of patient and physician compliance in drug administration (Smith *et al.*, 1979). It did not compare differences between prescribed and received doses of orally administered cytotoxic drugs. Failure in compliance may be a factor accounting for some relapses in childhood ALL, however specifically designed studies are needed to investigate this problem.

The overall randomised study of oral *vs* IM Mtx during continuing therapy of childhood ALL showed that IM Mtx produced no advantage in EFS and was associated with a greater incidence of neutrotoxicity, infectious complications and patients discomfort (Chessells *et al.*, 1987). Also IM Mtx did not appear to benefit any subgroup of patients. IM Mtx does produce higher peak concentrations and AUC, however there is no less variability than with oral Mtx. Although the unknown variable of patient compliance in drug administration is overcome with the administration by this route, both the higher serum concentrations and certainty of administration were not associated with improved leukaemia relapse free survival in this trial.

The mere achievement of higher serum concentrations of Mtx in the treatment of childhood ALL is not sufficient to improve the rate of cure in this disease. The relationship of Mtx and 6-MP dosage with EFS may indicate that those children who receive higher drug doses at levels as near as possible to those which are maximally tolerable have the greatest chance of cure of their disease.

Jeanette Stevens for data collection; the Leukaemia Research Fund for support of DRL; the North of England Children's Cancer Research Fund for support of ADJP and HA.

### References

BALIS, F.M., SAVITCH, J.L. & BLEYER, W.A. (1983). Pharmacokinetics of oral methotrexate in children. *Cancer Res.*, **43**, 2342.

BONADONA, G. & VALAGUSSA, P. (1981). Dose-response effect of adjuvant chemotherapy in breast cancer. *N. Engl. J. Med.*, **304**, 10.

CARDE, P., MACKINTOSH, F.R. & ROSENBERG, S.A. (1983). A dose and time response in the treatment of Hodgkin's disease with MOPP chemotherapy. *J. Clin. Onc.*, **1**, 146

CHABNER, B.A., ALLEGRA, C.J., CURT, G.A. & 5 others (1985). Polyglutamation of methotrexate. Is methotrexate a prodrug? *J. Clin. Invest.*, **76**, 907.

CHESSELLS, J.M., LEIPER, A.D., TIEDMAN, K., HARDISTY, R.M. & RICHARDS, S. (1987). Oral methotrexate is as effective as intramuscular methotrexate in maintenance therapy of childhood acute lymphoblastic leukaemia. *Arch. Dis. Child.*, **62**, 172.

CRAFT, A.W., RANKIN, A. & AHERNE, W. (1980). Methotrexate absorption in children with acute lymphoblastic leukaemia. *Cancer Treat. Rep.*, **S1**, 65, 77.

EVANS, W.E., CROM, W.R., STEWART, C.F. & 4 others (1984). Methotrexate systemic clearance influences the probability of relapse in children with standard risk acute lymphocytic leukaemia. *Lancet*, **i**, 359.

EVANS, W.E., CROM, W.R., ABROMOWITCH, M. & 5 others (1986). Clinical pharmacodynamics of high-dose methotrexate in acute lymphoblastic leukaemia – identification of a relation between concentration and effect. *New Engl. J. Med.*, **314**, 471.

EVANS, W.E., CROM, W.R., SCHELL, M.J., KALWINSKY, D.K. & RIVERA, G.K. (1989). Reappraisal of methotrexate clearance as a prognostic factor in childhood acute lymphocytic leukaemia. *Proc. Amer. Assoc. Canc. Res.*, **30**, 241 (957).

FREEMAN NARROD, M. (1962). Pharmacology of methotrexate. In *Methotrexate in the Treatment of Cancer*, Porter & Wiltshaw (eds) p. 17. Williams & Wilkins: Baltimore.

GAYNON, P., BLEYER, W.A., STEINHERZ, P. & 5 others (1987). Impact of treatment dose and delay on the disease free survival of children with acute lymphoblastic leukaemia and unfavourable prognostic features. *Proc. ASCO*, **6**, 156.

GUSHAW, J.B. & MILLER, J.G. (1978). Homogenous enzyme immunoassay for methotrexate in serum. *Clin. Chem.*, **24**, 1032.

HIYNIUK, W. & BUSH, H. (1984). The importance of dose intensity in chemotherapy and metastatic breast cancer. *J. Clin. Onc.*, **2**, 1281.

KEARNEY, P.J., LIGHT, P.A., PREECE, A. & MOTT, M.G. (1979). Unpredictable serum levels after oral methotrexate in children with acute lymphoblastic leukaemia. *Cancer Chemother. Pharmacol.*, **3**, 117.

LENNARD, L., LILLEYMAN, J.S. VAN LOON, J.V. & WEINSHILBOUM, R.M. (1990). Genetic variation in response to 6-mercaptopurine for childhood acute lymphoblastic leukaemia. *Lancet*, **i**, 336, 225.

LENNARD, L., REES, C.A., LILLEYMAN, J.S. & MADDOCKS, J.L. (1983). Childhood leukaemia: a relationship between intracellular 6-mercaptopurine metabolism and neutropaenia. *Br. J. Clin. Pharmacol.*, **16**, 359.

MEDICAL RESEARCH COUNCIL WORKING PARTY ON LEUKAEMIA IN CHILDHOOD (1986). Improvement in treatment for children with acute lymphoblastic leukaemia. *Lancet*, **i**, 408.

PEARSON, A.D.J., MILLS, S., AMINEDDINE, H.A., LONG, D.R., CRAFT, A.W. & CHESSELLS, J.M. (1987). Pharmacokinetics of oral and intramuscular methotrexate in children with acute lymphoblastic leukaemia. *Cancer Chemother. Pharmacol.*, **20**, 243.

PINKEL, D., HERNANDEZ, K., BORELLA, L. & 4 others (1971). Drug dosage and remission duration in childhood lymphocytic leukaemia. *Cancer*, **27**, 247.

PINKERTON, C.R., WELSHMAN, S.G., DEMPSEY, S.I., BRIDGES, J.M. & GLASGOW, J.F.T. (1980). Absorption of methotrexate under standardised conditions in children with acute lymphoblastic leukaemia. *Br. J. Cancer*, **42**, 613.

SILBERMAN, T., ROBISON, L.L., NESBIT, M.E., SATHER, H.N., ORTEGA, J.A. & HAMMOND, G.D. (1985). Association between outcome in childhood acute lymphoblastic leukaemia (ALL) and the amount of maintenance 6-Mercaptopurine. *Proc. ASCOs*, **4**, 166.

SMITH, S.D., ROSEN, D., TRUEWORTHY, R.C. & LOWMAN, J.T. (1979). A reliable method for evaluating drug compliance in children with cancer. *Cancer*, **43**, 169.

WHITEHEAD, V.M., ROSENBLATT, D.S., VUCHICH, M.J. & SHUSTER, J.J. (1990). Accumulation of methotrexate and methotrexate polyglutamates in lymphoblasts at diagnosis of childhood acute lymphoblastic leukaemia: A pilot prognostic factor analysis. *Blood*, **76**, 44.